# The symbiotic bacterial surface factor polysaccharide A on *Bacteroides fragilis* inhibits IL-1β-induced inflammation in human fetal enterocytes via toll receptors 2 and 4

**DOI:** 10.1371/journal.pone.0172738

**Published:** 2017-03-09

**Authors:** Fei Jiang, Di Meng, Meiqian Weng, Weishu Zhu, Wenxue Wu, Dennis Kasper, W. Allan Walker

**Affiliations:** 1 Laboratory of Rapid Diagnostic Technology for Animal Diseases, College of Veterinary Medicine, China Agricultural University, Beijing, China; 2 Mucosal Immunology and Biology Research Center, Massachusetts General Hospital *for* Children and Harvard Medical School, Charlestown, Massachusetts, United States of America; 3 Department of Microbiology and Molecular Genetics, Harvard Medical School, Boston, Massachusetts, United States of America; Istituto di Ricovero e Cura a Carattere Scientifico Centro di Riferimento Oncologico della Basilicata, ITALY

## Abstract

Colonizing bacteria interacting with the immature, unlike the mature, human intestine favors inflammation over immune homeostasis. As a result, ten percent of premature infants under 1500 grams weight develop an inflammatory necrosis of the intestine after birth, e.g., necrotizing enterocolitis (NEC). NEC is a major health problem in this population causing extensive morbidity and mortality and an enormous expenditure of health care dollars. NEC can be prevented by giving preterm infants their mother’s expressed breast milk or ingesting selective probiotic organisms. Vaginally delivered, breast fed newborns develop health promoting bacteria (“pioneer” bacteria) which preferentially stimulate intestinal host defense and anti-inflammation. One such “pioneer” organism is *Bacteroides fragilis* with a polysaccharide (PSA) on its capsule. *B*. *fragilis* has been shown developmentally in intestinal lymphocytes and dendritic cells to produce a balanced T-helper cell (TH1/TH2) response and to reduce intestinal inflammation by activity through the TLR2 receptor stimulating IL-10 which inhibits IL-17 causing inflammation. No studies have been done on the role of *B*. *fragilis* PSA on fetal enterocytes and its increased inflammation. Accordingly, using human and mouse fetal intestinal models, we have shown that *B*. *fragilis* with PSA and PSA alone inhibits IL-1β-induced IL-8 inflammation in fetal and NEC intestine. We have also begun to define the mechanism for this unique inflammation noted in fetal intestine. We have shown that *B*. *fragilis* PSA anti-inflammation requires both the TLR2 and TLR4 receptor and is in part mediated by the AP1 transcription factor (TLR2) which is developmentally regulated. These observations may help to devise future preventative treatments of premature infants against NEC.

## Introduction

Colonizing bacteria interacting with the immature human intestine, unlike the mature intestine, favors inflammation over homeostasis [1,2]. As a result ten percent of premature infants develop an inflammatory necrosis of the intestine after birth, a condition known as necrotizing enterocolitis (NEC) [3,4]. NEC is a major health problem in this patient population causing extensive morbidity and mortality and an enormous expenditure of health care dollars [5].

Yet certain commensal bacteria used as probiotics, when given to newborn prematures can prevent or lessen the severity of NEC [6,7]. What is it about the immature intestine interacting with colonizing bacteria that causes this paradoxical response? We hypothesize that commensal bacteria interacting with immature enterocytes can result in either an excessive inflammatory response or an inhibition of inflammation depending on the composition of the immature signaling pathways in the enterocyte. Support for this hypothesis, as published data, exists from this laboratory [1,4,8]. We have identified that the genes responsible for the innate immune response pathway, e.g., NFκB, IL-8, IL-6, and their negative regulators (SIGRR, IRAK-M, A20, etc.) are either increased (NFκB, IL-8, IL-6) or decreased (negative regulators SIGRR, IRAK-M, A20, etc.) developmentally causing an aberrant response to these bacteria [8–10]. We have also shown that a secreted factor from *Bifidobacterium infantis* (a probiotic preventative of NEC) preferentially downregulates an IL-1β inflammatory stimulus in fetal enterocytes [4,11]. These observations suggest that immature enterocytes interacting with commensal bacteria or their secretions have unique inflammatory or anti-inflammatory pathways.

In fullterm neonates, an appropriate initial bacterial colonization of the intestine is necessary for normal development of immune defense leading to homeostasis [12]. Optimal colonization occurs when a full term infant is born vaginally, receiving maternal vaginal/colonic microbiota. The initial bolus of maternal microbiota is further stimulated by oligosaccharides and other active factors (pIgA, lactoferrin, etc.) in breast milk which activate proliferation of so called “pioneer” bacteria, essential for stimulus of gut development [13]. Such “pioneer” bacteria isolated from newborn gut have been shown to stimulate the development of sIgA production [14] and to reduce excessive intestinal inflammation [15].

One such “pioneer” bacterium noted in infant intestine early in life is *Bacteroides fragilis* [13]. This bacterium expresses on its surface a polysaccharide (PSA) which has been shown to affect the development of immune function [14] in macrophages and dendritic cells. Polysaccharide A (PSA) creates a balanced TH1/TH2 helper cell response which may prevent the development of allergy [15]. PSA also interacts with TLR2 receptor molecules on CD4 lymphocytes to preferentially stimulate T-regulatory cells to produce IL-10 [16] which prevents experimental induced colitis via IL-17 stimulation [17,18].

To date no studies have examined the effect *B*. *fragilis* PSA on enterocytes. Since this organism appears early in the newborn intestine and is anti-inflammatory, we studied its effect on fetal human enterocytes as a possible basis for prevention of NEC. In this study, we used *ex vivo* models of human fetal and NEC intestine and fetal mouse intestine to determine the mechanisms of *B*. *fragilis* PSA inhibition of IL-1β stimulated inflammation.

## Materials and methods

### *Bacteroides fragilis* and polysaccharide A (PSA)

*B*. *fragilis* NCTC 9343 (dossa) containing PSA on its capsule and a mutant strain without PSA (delta) were provided by Dr. Dennis Kasper, Department of Microbiology and Molecular Genetics, Harvard Medical School. These strains have been previously described in detail [19]. The organisms were grown anaerobically in broth culture as previously described [20] until they reached a stationary phase of growth (OD 600>1.0). Initially, H4 cells, a human fetal nontransformed primary small intestinal cell line characterized by our laboratory **[21]**, were incubated with either wildtype *B*. *fragilis* (PSA dossa) or mutant *B*. *fragilis* (PSA delta) at 10^7^ or 10^8^ organisms per cc in media for one hour. H4 cells were then washed with PBS and incubated in the medium with gentamicin (100 μg/ml) for one hour, washed with PBS then stimulated with of IL-1β (5 ng/ml) for 24 hrs in the media containing 10 μg/ml gentamicin to kill extracellular organisms after initial interaction. The supernatants were then saved for ELISA analysis in a -20°C refrigerator.

Purified PSA was also provided by Dr. Dennis Kasper. PSA has previously been isolated, purified and characterized from *B*. *fragilis* NCTC 9343 as reported **[22]**. A dose response curve was obtained for the optimal effect of PSA on H4 cells and on organ cultures of fetal mouse intestine. H4 cells were incubated with purified PSA (400 μg/cc media) for 24 hrs before exposure to IL-1β (5 ng/ml) for 24 hrs. Supernatants were saved at -20°C for ELISA assay.

### H4 cell line subjected to TLR2 and TLR4 siRNA transfection

H4 cells, were cultured in Dulbeco’ modified Eagle’s medium (DMEM) (Gibco Thermofisher, Woburn, MA) with 10% fetal bovine serum (FBS) (Mediatech, Manassa, VA), 0.2 u/ml insulin (Eli Lilly and Company, Indianapolis, IN), 2 mM L-glutamine, 0.1 mM MEM nonessential amino acids, 10 mM Hepes buffer, 100 units/ml penicillin and 100 μg/ml streptomycin (all purchased from Gibco Thermofisher). H4 cells were transfected with stealth human TLR2 or TLR4 siRNA or control siRNA following the manufacturer’s instructions (Invitrogen Thermofisher, Grand Island, NY). Briefly, for each well of 6-well plates to be transfected, RNAi and Lipofectamine RNAiMAX complexes were prepared as follows: 25 pmol of diluted RNAi in 400 μl Opti-MEM I medium without serum; 4 μl of Lipofectamine RNAiMAX was added to each well containing the diluted RNAi molecules and incubated for 20 minutes at room temperature. 3x10^5^ H4 cells were added in 600 μl of antibiotic free H4 growth media and then added to each well resulting in 25nM RNAi in the culture. After 24 hrs of incubation at 37°C, an additional 1 ml of H4 growth media without antibiotics was added to each well resulting in a 12.5 nM final concentration. All transfection reagents were purchased from Invitrogen Thermofisher. Efficiency was analyzed by real time qRT-PCR and red oligo staining 72 hrs after the transfection (the knock-down rate was 71.6% TLR4, 94% TRL2 and transfection-rate was 94.8%). The cells were incubated with PSA control media for 24 hrs beginning at 48 hrs after transfection and then exposed or not to 1ng/ml of recombinant human IL-1β (R&D Systems, Minneapolis, MN)) for 24 hrs The supernatants at 24 hrs were collected and stored at −20°C for an enzyme-linked immunosorbent assay (ELISA) analysis.

### NEC enterocyte experiments

NEC-IEC were isolated and cultured from the viable margins of resected ileal NEC tissue from a NEC neonate at 25-wk gestation [4,11]. NEC-IEC were cultured in Opti-Minimal Essential Medium (Opti-MEM) (Gibco Thermofisher) supplemented with 10% heat-inactivated FBS (Mediatech), 0.2 U/ml insulin (Eli Lilly and Company), 20 ng/ml epidermal growth factor (EGF) (EMD Millipore, Billerica, MA), and a 1% antibiotic–antimycotic cocktail (Gibco Thermofisher) in a sterile cell culture humidifier at 32°C and 5% CO2. The cells were incubated with PSA or control PBS media for 24 hrs and then exposed or not to 1ng/ml of recombinant human IL-1β (R&D Systems, Minneapolis, MN) for 24 hrs. The supernatants were then collected and stored at −20°C for a later ELISA analysis. Access to NEC discarded tissue was approved by a Partners IRB (Protocol #:1999P003833/MGH).

### Fetal mouse model

C57BL/6J wild type (WT) mice and TLR4^-/-^ and TLR2^-/-^ mice, both on a C57BL/6J background (Jackson Laboratory), were used. All mice were bred and housed in a specific pathogen free facility. Animals were given water and standard laboratory chow *ad libitum*. Timed pregnant mice were established by pairing 10–12-wk old female mice with proven breeder males just prior to the end of the daily light cycle. The following morning, each female was examined for the presence of an ejaculatory plug in the vagina. When noted, the female was placed in a dated cage and considered pregnant, e.g., embryonic day (E) 0.5. Pups were delivered by caesarean section at E 18.5 [11]. Ileal tissues were then collected for experiments. Animal procedures had been previously approved by the Massachusetts General Hospital Subcommittee on Research Animal Care and Use committee (A3596-01).

### Fetal mouse ileal organ culture

WT, TLR4^-/-^ and TLR2^-/-^ mouse fetal ileal tissues harvested at E 18.5 were cut into 3-mm pieces and maintained in organ culture media as described **[11]** but also supplemented with an antibiotic-antimycotic cocktail (100 unit/ml penicillin, 100 μg/ml streptomycin and 0.25 μg/ml fungizone antimycotic) (GibcoThermofisher). After 2 h at 37°C, tissues were pre-treated with and without PSA for 24 hrs and then stimulated with 5ng/ml of recombinant mouse IL-1β (R&D Systems) for 24 hrs Supernatants were collected and stored at −20°C for ELISA analysis.

### ELISA

Levels of IL-8 were measured in culture supernatants using ELISA kits (R & D system) according to the manufacturer’s instructions. IL-8 was quantified in each supernatant in triplicate. Colorimetric results were read at a wavelength of 450 nm. Values were normalized to total protein in cells or organ cultures. Protein was determined by a bicinchoninic acid protein assay (Pierce, Rockford, IL) modified for 96-well microtiter plates according to the manufacturer’s protocol.

### Immunofluorescent staining

H4 cells cultured on Falcon Culture Slides (Thermofisher) were transfected with human stealth TLR2 or TLR4 siRNA or control siRNA and then pre-treated with PSA for 24 hrs as described above and stimulated with IL-1β for 2 hrs The cells were washed with phosphate buffered saline (PBS) (Gibco Thermofisher) once and fixed in 4% paraformaldehyde in PBS for 15 minutes at room temperature (RT). Cells were washed three times with PBS to remove paraformaldehyde and then blocked in blocking buffer (1xPBS / 3% bovine serum albumin/0.3% Triton X-100) for 60 minutes at RT. The cells were then incubated with either anti-phospho-c-Jun (P-c-Jun) (1:80) or anti-phospho-c-Fos (P-c-Fos; 1:800) in antibody dilution buffer (1xPBS / 1% BSA / 0.3% Triton X-100) overnight at 4°C. After being washed three times with PBS, both were stained with goat anti-rabbit fluorescein isothiocyanate (FITC) (1:500) at RT for 60 minutes and washed again with PBS three times. The cell membrane was then stained by anti-phospholipid antibody conjugated with rhodamine (1:500) for 1 hour at RT. Specimens were then washed three times with PBS, mounted with ProLong Antifade Reagent (Life Technologies) and analyzed with a fluorescent Nikon confocal microscope. The fluorescent intensity was analyzed with Image J (fiji-win64) software. Corrected Total Cell Fluorescence (**CTCF)** = **Integrated Density—(Area of selected cell** X **the mean fluorescence of background readings)** [23] and then calculated.

### Real-time quantitative reverse transcription PCR

The RNA RNeasy Mini kit (Quigen, Valencia, CA) was used for extraction of total RNA from H4 cells. RNA was reverse transcribed with random hexamers using an Advantage RT-for–PCR kit (Clontech, Mountain View, CA). The cDNA was amplified using iQ SYBR Green Supermix (Bio-Rad, Philadelphia, PA) and 500nM of each primer specified in the next paragraph. β-actin primers were amplified in all samples. Triplicate cDNA samples were amplified with the following primers: Mean threshold cycle (CT) values of each transcript were normalized by subtracting the mean CT value for the β-actin transcript of that sample. The change in the normalized transcript level was expressed relative to the control sample with a change of *n* in CT representing a 2*n* or greater -fold difference, as described previously [24]. Primer sequences used in this study were: *Human-TLR2*, forward *5’-* ATCCTCCAATCAGGCTTCTCT -3’, and reverse, 5’—GGACAGGTCAAGGCTTTTTACA; *Human-*TLR4 forward, 5’- ATGCTGCCGTTTTATCACGGA—3’ and reverse, 5’- CTAAACTCTGGATGGGGTTTCC– 3’, *Human*-*β-actin*, forward, 5’- CATGTACGTTGCTATCCAGGC-3’, and reverse, 5’- CTCCTTAATGTCACGCACGAT-3’

### Statistical analysis of data

Results were expressed as the mean ± standard error of the means (SEM). One-way analysis of variance (ANOVA) was used to compare the mean of multiple groups. Differences of a *P* value <0.05 were considered significant (*P < 0.05, **P < 0.01, ***P < 0.001) (GraphPad Prism 6).

## Results

### *B*. *fragilis* with PSA was anti-inflammatory with H4 cells

**Fig 1:**
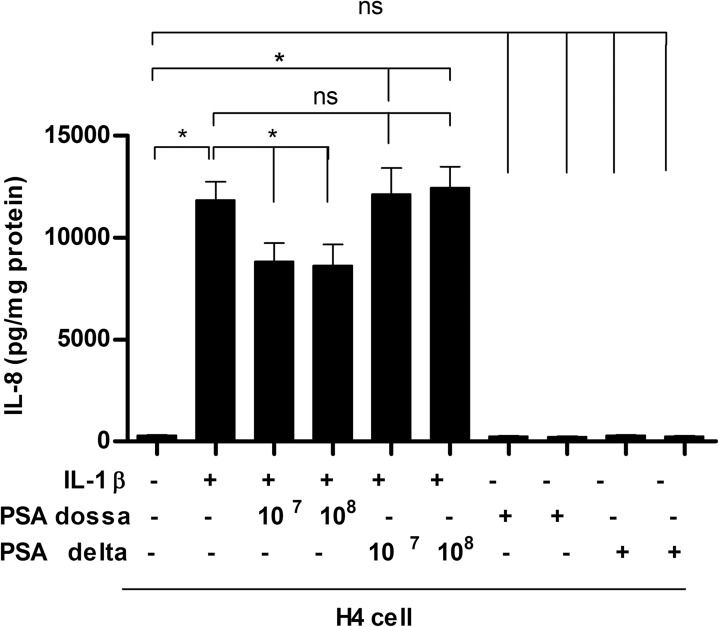
Wild type (WT) *B*. *fragilis* with polysaccharides A (PSA dossa) reduced IL-1 β triggered IL8 in immature human fetal intestinal epithelial cell line (H4 cells). H4 cells were pretreated with WT *B*. *fragilis* PSA (dossa) or mutant type without PSA (delta) at an indicated dosage for 1 hour. Then the cells were stimulated with or without IL-1β for 24 hrs. The supernatant IL8 levels were determined by Elisa. Data are represented as mean ± SD (n = 3, *P< 0.05, one-way ANOVA and post-hoc tests). Three experiments with similar results were analyzed.

In order to determine the effect of PSA expressed on the surface of B. *fragilis* on anti-inflammation after IL-1β stimulus, H4 cells were incubated with either wild type *B*. *fragilis* (PSA dossa) or mutant *B*. *fragilis* (PSA delta) followed by an inflammatory stimulus of IL-1β or not. Only *B*. *fragilis* expressing PSA on its surface resulted in a reduction in the IL-8 response (Fig 1)

### Purified PSA was anti-inflammatory with H4 cells

**Fig 2:**
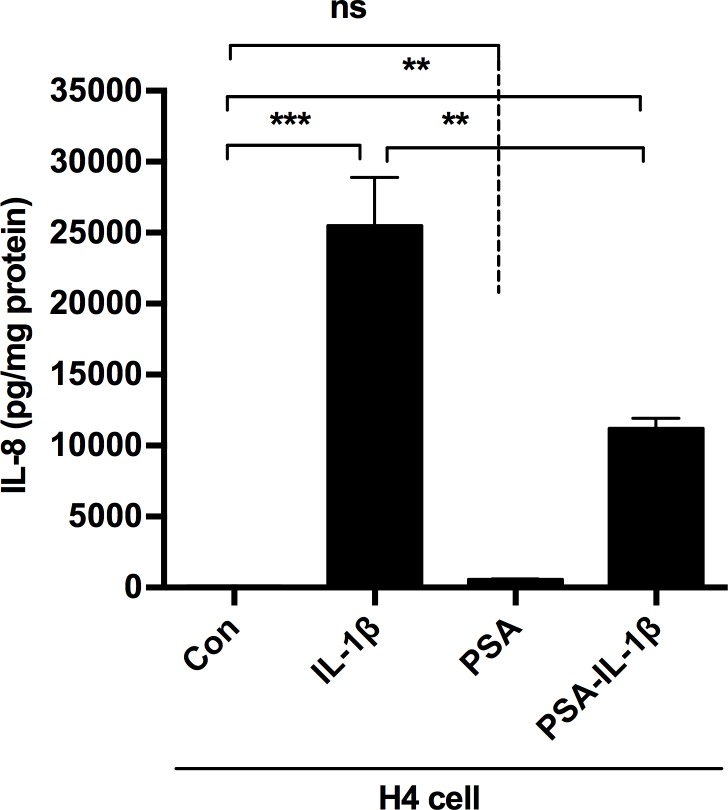
PSA reduced IL-1βinduced IL-8 in H4 cells. H4 cells were pretreated with or without PSA at an optimal dose and then exposed to IL-1β or treated with PSA alone. The supernatants were collected to measure IL8 levels by Elisa. The mean and SEM were from triplicate wells and were representative of three separate experiments with similar results. **P<0.01 (one-way ANOVA and post-hoc tests).

We next used purified PSA exposed to H4 cells to begin to define the anti-inflammatory effect. When purified PSA was exposed to H4 cells at the optimal dose, based on a dose response curve, before an IL-1β inflammatory stimulus or not. IL-8 was significantly reduced compared to controls (Fig 2).

### PSA is anti-inflammatory in fetal enterocytes from resected NEC small intestine (NEC-IEC)

**Fig 3:**
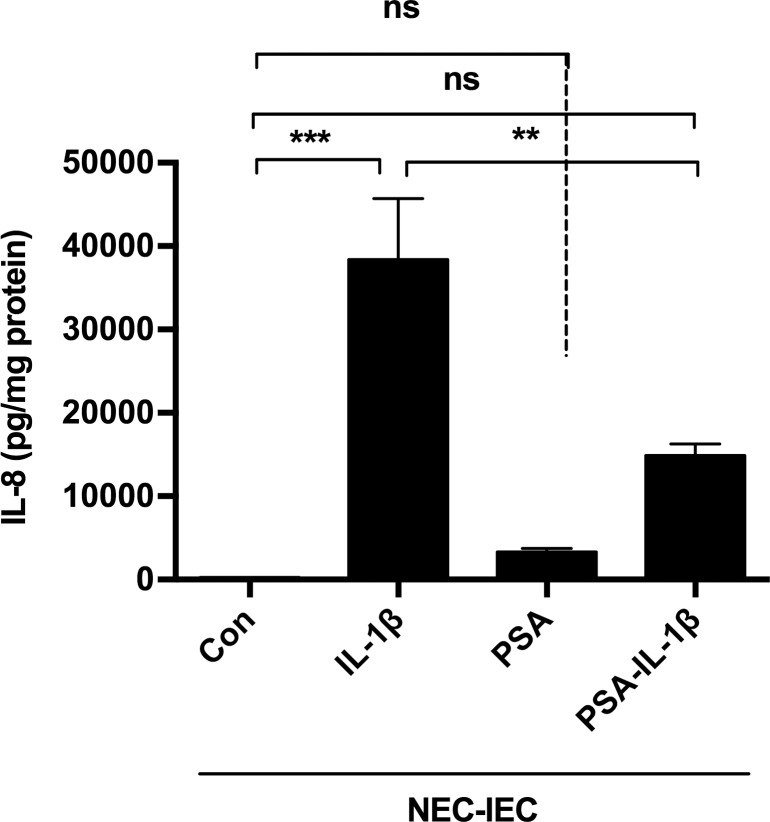
PSA reduced IL-1 β triggered IL-8 in necrotizing enterocolitis intestinal epithelial cells (NEC-IEC). NEC-IEC cells were pretreated with or without PSA and then exposed to IL-1 β for 24 hrs or treated with PSA alone. The supernatant IL8 levels induced by IL-1 β were reduced by PSA pretreatment. The mean and SEM were from triplicate wells and were representative of three separate experiments with similar results. **P<0.01, ***P<0.001(one-way ANOVA and post-hoc tests).

In order to confirm that the *B*. *fragilis-*PSA effect also existed in a primary cell line isolated from the resected small intestine of a NEC patient at surgery, NEC-IECs were incubated with PSA and then stimulated with IL-1β. As with H4 cells, PSA was anti-inflammatory in NEC-IECs (Fig 3).

### The PSA effect on H4 cells is TLR2 and TLR4 dependent

**Fig 4:**
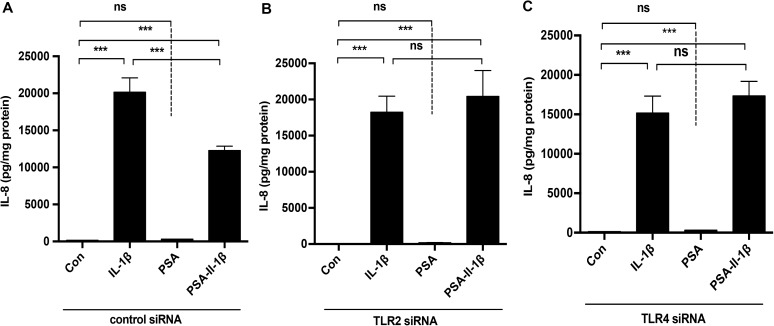
Both TLR4 and TLR2 are required for PSA prevention of IL-1β induced IL8 in H4 cells. Control(A) and TLR2(B) or TLR4 (C) knockdown H4 cells were pretreated with or without PSA and then exposed to IL-1β for 24 hrs or treated with PSA alone. The supernatants were collected to measure IL8 levels by Elisa. The mean and SEM were from triplicate wells and were representative of three separate experiments with similar results ***P<0.001(one-way ANOVA and post-hoc tests).

Having previously shown that “pioneer” bacterial secretions anti-inflammatory effect was TLR4 dependent and that *B*. *fragilis* PSA mediated anti-inflammation in dendritic/lymphoid cells, we begin to determine the mechanism of the PSA effect on H4 cells, TLR2 and TLR4 molecules were knocked down. Control cells and knocked down cells were exposed to PSA before IL-1β inflammatory stimulus. An anti-inflammatory effect was only noted in control H4 cells and not in TLR2 or TLR4 knocked down cells. (Fig 4).

### PSA was not anti-inflammatory in TLR2 and TLR4 knockout fetal mouse small intestinal organ culture

**Fig 5:**
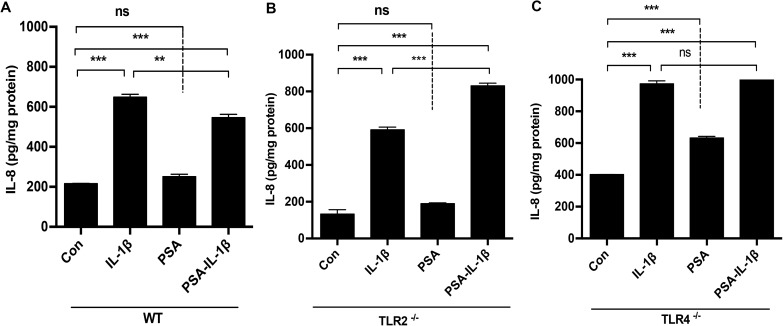
TLR4 and TLR2 are required for PSA reduction of IL-1β-induced IL8 in fetal mouse small intestinal organ cultures. Embryonic 18.5 day (E18.5) mouse fetal small intestinal organ culture were pretreated with or without PSA in (A) wild type (WT), (B) TLR2^-/-^and (C) TLR4^-/-^ mice. The supernatant IL8 levels were detected 24 hrs after IL-1 β stimulation. The mean and SEM were from triplicate wells and were representative of three separate experiments with similar results. **P<0.01, ***P<0.001(one-way ANOVA and post-hoc tests).

To confirm the *in vitro* observation that TLR2 and TLR4 were involved in anti-inflammation in H4 cells, in vivo TLR2^-/-^ and TLR4^-/-^ mice were used in organ culture to determine the PSA effect an IL-1β inflammatory stimulus. TLR2 and TLR4 knockout fetal mice were exposed to PSA and stimulated with IL-1β. As with H4 cells, PSA was anti-inflammatory in control but not TLR2 or TLR4 knockout mice (Fig 5).

### PSA was effective in reducing IL-1β induced P-c-Jun expression in control and TLR4 knockdown but not TLR2 knockdown H4 cells

**Fig 6:**
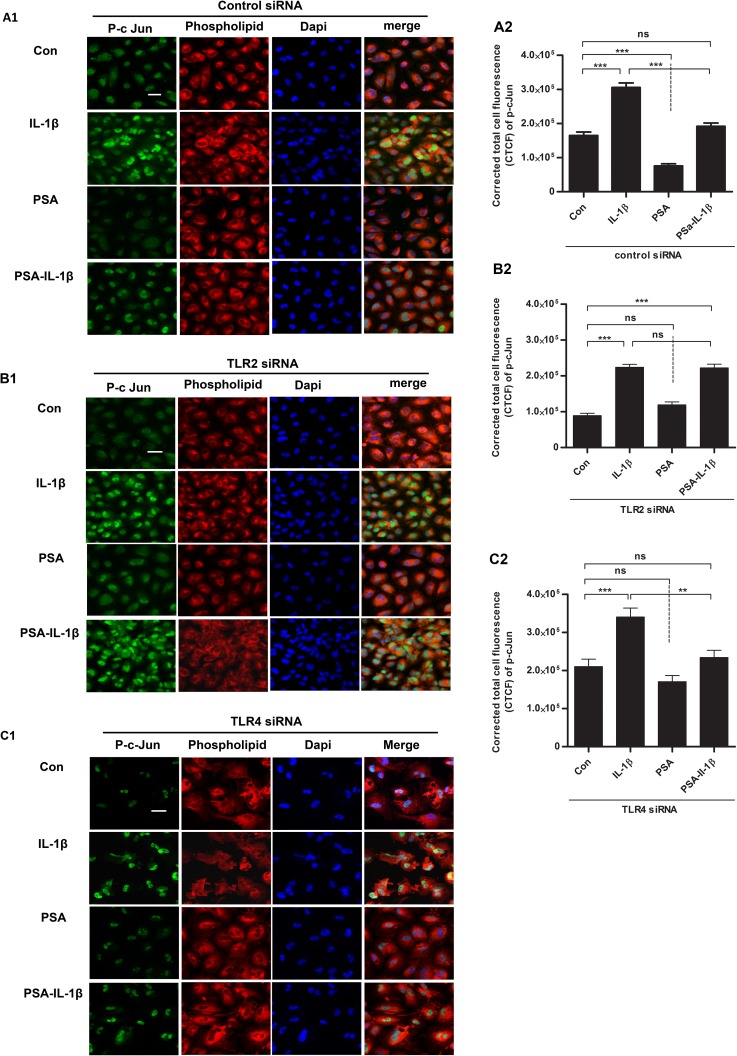
PSA reduced IL-1β induced P-c-Jun expression is TLR2 dependent. P-c-Jun (green color) was detected by immunofluorescent staining in control (A1), TLR2 knockdown (B1) and TLR4 knockdown (C1) H4 cells which were pretreated with or without PSA for 24 hrs and then exposed to IL-1β for 2 hrs The nuclei were counterstained by Dapi (blue), and the cell membrane was stained red. Amplified x600; scale bar = 20 μm; n = 60–70 cells/treatment. The images were representative of three separate experiments with similar results. The analysis (A2, B2 and C2) applied by corrected total cell fluorescence (CTCF) was determined.** P<0.01, ***P<0.001(one-way ANOVA and post-hoc tests).

To determine if AP1 transcription factor activation by IL-1β occurred and TLR2 or TLR4 knocked down H4 cells was involved after PSA exposure (Fig 6), control siRNA (Fig 6A1 and 6A2), TLR2 siRNA (Fig 6B1 and 6B2) and TLR4 siRNA (Fig 6C1 and 6C2) transfected cells were exposed to IL-1β with and without PSA pretreatment and P-c-Jun staining was determined. The results showed that P-c-Jun was increased significantly after IL-1β stimulation and that PSA reduced the response in control siRNA treated and TLR4 knocked down siRNA but not in TLR2 knockdown cells. The same results were noted for the P-c-Fos gene (data not shown). These results suggest that after TLR2 knockdown, the transcription factor genes (P-c-Jun) were activated in response to IL-1β. The response was inhibited after PSA exposure in part due to TLR2 but not TLR4 dependence.

## Discussion

This laboratory has had a long standing interest in the interaction between colonizing bacteria and the developing human intestine. We have shown that colonizing bacteria interacting with the premature human intestine preferentially stimulates inflammation over immune homeostasis [1,2,8]. We have also reported that the stimulation by certain bacteria can mediate excessive inflammation through activation of immature innate immune inflammatory response genes in fetal enterocytes [8,11]. Yet, there are also clinical and experimental evidence to suggest that probiotics and their secretions may also have a protective effect against this enhanced inflammatory response [6,25] suggesting a paradoxical situation. This observation may be important clinically. A major intestinal inflammatory condition, necrotizing enterocolitis (NEC), occurs commonly in premature infants less than 1500 gms [3,10].

NEC is associated with multiple risk factors after premature birth including hypoxic-ischemic events, the use of formula feeding, and a dysbiotic bacterial colonization resulting in an increased likelihood of severe intestinal inflammation, coagulation necrosis and gastrointestinal bleeding [3,5,12]. We believe that a major pathogenic mechanism of NEC is the accentuated response of the immature intestine to colonizing bacteria. Yet paradoxically a major probiotic which has been shown clinically to protect against the expression of NEC is *Bifidobacterium longum subsp infantis* [4,8,25]. Accordingly, we have carefully studied the mechanism by which this probiotic and its secretions alter enterocyte inflammation in the immature human intestine.

In that study, we focused on the effect of *B*. *infantis* secretions on the IL-1β-induced inflammatory response in premature human enterocytes. IL-1β is an important inflammatory cytokine associated with the pathogenesis of NEC and functions by mediating inflammatory cell recruitment and amplifying the innate immune response via enterocyte signaling [26,27]. The gut is colonized with trillions of beneficial commensal microorganisms which normally maintain a mutually beneficial symbiotic relationship with the host. To maintain intestinal homeostasis, both IEC and gut associated immune cells recognize bacterial components via pattern recognition receptors (PRRs) to conserve a balance between tolerance to the large communities of resident luminal bacteria while at the same time being able to mount an inflammatory response against pathogens [28]. TLRs are a major class of PRRs that are present on IECs and mucosal immune cells which are involved in the induction of both tolerance and inflammation [29,30].

TLR2 and TLR4 are two of 10 or more members of the TLR family. In recent studies with *B*. *fragilis* expressing PSA and with PSA alone, TLR2 had been implicated in the interaction with CD4 lymphocytes and dendritic cells [16,17]. *B*. *fragilis* with PSA has been identified during the first week of life in intestinal contents of full term, vaginally delivered human infants, particularly when the infant was exclusively breast fed [13]. This study is an initial in vitro attempt to identify with another so called “pioneer” colonizing bacterium, B. *fragilis*, if additional anti-inflammatory effects occur in a fetal intestinal cell line after inflammatory stimulus. This so called “pioneer” microorganism has been implicated in reducing the tendency for intestinal inflammation in infant intestine in the first month of life [4]. Experimentally, *B*. *fragilis* containing PSA and PSA alone when given to germ free mouse models stimulate a balance between TH1 and TH2 CD4 lymphocytes, a step thought to be necessary in developing of immune homeostasis and preventing the increased expression of allergic disease seen in developed countries [31]. TLR2 has been specifically implicated in the stimulation of FOXP3+ Tregs cells to produce IL-10 which leads to the suppression of the release of IL-17 cytokines implemented in intestinal inflammation [16]. In recent studies, we have shown that secretions from *Bifidobacteria infantis*, another “pioneer” bacteria, can mediate IL-1β-induced anti-inflammation in fetal intestine via the TLR4 receptor [11], suggesting that increased expression of TLR2 and TLR4 on the immature intestinal surface may help to mediate anti-inflammation from these “pioneer” neonatal bacteria. However, no studies have been done to determine the role of the “pioneer” expressed microorganism *Bacteroides fragilis* in preventing excessive enterocyte inflammation in human immature intestine. Accordingly, using established ex vivo human fetal intestinal models developed in this laboratory, we started to determine the effect of PSA on *Bacteroides fragilis* an initial in vitro study using a fetal intestinal primary cell line and primary enterocytes isolated from NEC intestinal resection.

We have previously reported that fetal human intestine favors excessive inflammation over anti-inflammation [8]. We have also reported that conventional commensal bacteria can trigger intestinal inflammation in fetal but not mature intestinal enterocytes, presumably due to expression of immature innate immune inflammatory response genes [32]. Yet premature infants can be protected from the severe intestinal inflammation of NEC by commensals acting as probiotics [6,7,25]. This paradoxical response to colonizing bacteria by fetal intestine was recently underscored by our published observation that secretions from another “pioneer” bacterium (*Bacteroides infantis*) can reduce intestinal inflammation after an IL-1β inflammatory stimulus. Furthermore, as stated this anti-inflammatory response by *B*. *infantis* secretions is mediated through the TLR4 receptor [11]. This observation represents an unconventional function for the TLR4 receptor and suggests that toll receptors in the immature intestine may exhibit different functions.

Accordingly, since the TLR2 receptor has been implicated in the function of *B*. *fragilis* PSA and the development of immune homeostasis, particularly anti-inflammation of lymphocyte cells during the newborn period and *B*. *infantis* mediates anti-inflammation through the TLR4 receptor, we have investigated the role of *B*. *fragilis* PSA in excessive inflammation by fetal human enterocytes and have begun to determine the role of TLR2 and TLR4in this process.

Therefore, we have used a human fetal small intestinal epithelial cell line (H4 cells) and knocked down its TLR2 and TLR4 genes. The cells were then pretreated with or without PSA and stimulated with IL-1β before measuring IL-8. The results showed that *B*. *fragilis* with PSA and PSA alone prevented IL-8 induction in response to IL-1β in control but not in TLR2 and TLR4 gene knockdown cells. These data suggest that both TLR2 and TLR4 are participants in the *B*. *fragilis* PSA effect in the prevention of the IL-8 inflammatory response in immature H4 cells. We have demonstrated a similar PSA anti-inflammatory effect in NEC-IEC. To confirm these results, we extended these observations to an *in vivo* fetal mouse model by applying similar conditions to embryo 18.5 day (E 18.5) fetal small intestinal organ cultures [11] from control, TLR4^-/-^ and TLR2^-/-^ mouse strains. PSA attenuated IL-8 induction in response to IL-1β in control but not in TLR2^-/-^ and TLR4^-/-^mice. These results suggest that PSA requires both the TLR2 and TLR4 receptors to prevent or attenuate IL-1β—induced IL-8 activation. Although the underlying mechanism for anti-inflammation via both TLR2 and TLR4 is not known, we do know that PSA is a zwitterion with both positive and negative charges expressed on the molecule [20,33]. We also know that TLR2 and TLR4 can interact with both positive and negative charged molecules [34,35]. Accordingly, electrostatic interaction with surface molecules, e.g., TLRs on the fetal enterocyte may contribute to a decrease in signals that mediate the IL-8 response. Further studies are needed however to establish the actual mechanism, particularly for the TLR4 receptor.

We have previously reported that AP-1 is a specific nuclear transcriptional factor for IL-1β induced IL-6/IL-8 secretions, but only in immature enterocytes [36]. AP-1 is composed of c-Jun and c-Fos genes which are phosphorylated in their active form. To investigate the role of AP-1 in probiotic prevention of IL-1β-induced IL-8 in H4 cells, we used TLR2 and TLR4 gene knocked down cells and pretreated these cells with or without PSA before stimulation with IL-1β. Immunofluorescent staining was used to determine phospho-c-Jun (P-c-Jun) and phospho-c-Fos (P-c-Fos) (data not shown). In this study, PSA exposure prevented both the P-c-Jun and P-c-Fos increase in WT and TLR4 knockdown cells but not in TLR2 knockdown cells. These observations suggested that in immature enterocytes *B*. *fragilis* with PSA and PSA alone prevent IL-1β signaling in TLR2 knockdown but not TLR4 knockdown at least in part through AP-1 genes. Although TLR4 is involved in anti-inflammation it functions in immature enterocytes by as yet to be determined additional mechanisms. We plan to examine transcription factors of PSA interaction with H4 cell RNA to determine possible additional pathways for TLR4 involvement in PSA-mediated anti-inflammation in fetal and NEC enterocytes.

## Conclusions

This study shows for the first time that *Bacteroides fragilis* with PSA and PSA alone has an anti-inflammatory effect on IL-1β stimulation of IL-8 in fetal human enterocytes, NEC enterocytes and fetal mouse small intestine. This anti-inflammation is both TLR2 and TLR4 dependent and mediated in part through a down regulation of P-c-Jun and P-c-Fos of the AP1 (TLR2 response), a developmentally-regulated transcription factor. This in vitro study represents an initial step in defining the role of B. *fragilis* PSA in anti-inflammation in the premature intestine. Before recommendations can be made for clinical use of this “pioneer” bacterium, the observation needs to be repeated in a mouse NEC model and decreased inflammation demonstrated after B. *fragilis* PSA and mutant non PSA (not demonstrated) strands have been studied. In addition, we plan to confirm these results of this organism’s effect in TLR2^-/-^ and TLR4^-/-^ premature neonates. Since *B*. *fragilis* with PSA is a “pioneer” bacterium seen in the neonatal intestine, particularly with breastfeeding, this observation provides a further basis for promoting use of expressed breastmilk in premature infants, a known preventative measure to prevent NEC. In future studies, PSA could also be used in a clinical trial as a “pioneer” bacterial product for premature infants to prevent NEC.

## Supporting information

S1 File Individual data points used to build all the figures. (PDF)

## References

[1] Nanthakumar NN, Fusunyan RD, Sanderson I, Walker WA. Inflammation in the developing human intestine: A possible pathophysiologic contribution to necrotizing enterocolitis. Proc Natl Acad Sci USA 2000; 97:6043–6048. 10823949 10.1073/pnas.97.11.6043 PMC18555

[2] Nanthakumar NN, Young C, Ko JS, Meng D, Chen J, Buie T, et al Glucocorticoid responsiveness in developing human intestine: possible role in prevention of necrotizing enterocolitis. Am J Physiol Gastrointest Liver Physiol 2005; 288:G85–92. 10.1152/ajpgi.00169.2004 15591589

[3] Neu J, Walker WA. Necrotizing enterocolitis. N Engl J Med 2011; 364:255–264. 10.1056/NEJMra1005408 21247316 PMC3628622

[4] Ganguli K, Meng D, Rautava S, Lu L, Walker WA*, Nanthakumar N*. Probiotics prevent necrotizing enterocolitis by modulating enterocyte genes that regulate innate immune-mediated inflammation. Am J Physiol Gastrointest Liver Physiol 2013; 304:G132–141. (*Shared senior authorship) 10.1152/ajpgi.00142.2012 23139215 PMC3543644

[5] Lin PW, Stoll BJ. Necrotizing enterocolitis. Lancet 2006; 368:1271–1283. 10.1016/S0140-6736(06)69525-1 17027734

[6] Alfaleh K, Anabrees J. Probiotics for prevention of necrotizing enterocolitis in preterm infants. Cochrane Database Syst Rev 2014; 4:CD005496. 10.1002/14651858.CD005496.pub4 24723255

[7] Alfaleh K, Anabrees J. Probiotics for prevention of necrotizing enterocolitis in preterm infants. Evid Based Child Health 2014; 9:584–671. 10.1002/ebch.1976 25236307

[8] Nanthakumar N, Meng D, Goldstein AM, Zhu W, Lu L, Uauy R, et al The mechanism of excessive intestinal inflammation in necrotizing enterocolitis: an immature innate immune response. PLoS One 2011; 6:e17776 10.1371/journal.pone.0017776 21445298 PMC3061868

[9] Nanthakumar NN, Klopcic CE, Fernandez I, Walker WA. Normal and glucocorticoid-induced development of the human small intestinal xenograft. Am J Physiol Regul Integr Comp Physiol 2003; 285:R162–170. 10.1152/ajpregu.00721.2001 12560204

[10] Grave GD, Nelson SA, Walker WA, Moss RL, Dvorak B, Hamilton FA, et al New therapies and preventive approaches for necrotizing enterocolitis: report of a research planning workshop. Pediatr Res 2007; 62:510–514. 10.1203/PDR.0b013e318142580a 17667844

[11] Meng D, Zhu W, Ganguli K, Shi H, Walker WA. Anti-inflammatory effects of *Bifidobacterium longue subsp infantis* secretion on fetal human enterocytes are mediated by TLR4 receptors. Articles in PresS. Am J Physiol Gastrointest Liver Physiol 2016; (August 25, 2016). 10.1152/ajpgi.00090.2016 PMC5142200 27562058

[12] Houghteling P, Walker WA. Why is initial bacterial colonization of the intestine important to the infant’s and child’s health. J Pediatr Gastro Nutr 2015; 60:294–307. 10.1097/MPG.0000000000000597 PMC4340742 25313849

[13] Jost T, Lacroix C, Breagger C, Chassard C. New insights in gut microbiota establishment in healthy breast fed neonates. PLoS One 2012; 7:e44595–603. 10.1371/journal.pone.0044595 22957008 PMC3431319

[14] Sjogren YM, Tomicic S, Lundberg A, Bottcher MF, Bjorksten B, Sverremark-Ekstrom E, Jenmalm MC. Influence of early gut microbiota on the maturation of childhood mucosal and systemic immune response. Clin Exp Allergy 2009; 39:1842–1851. 10.1111/j.1365-2222.2009.03326.x 19735274

[15] Mazmanian DK, Liu CH, Tzianabos AO, Kasper DL. An immunomodulatory molecule of symbiotic bacteria directs maturation of the host immune system. Cell 2005; 122:1070–118. 10.1016/j.cell.2005.05.007 16009137

[16] Round JL, Lee SM, Li J, Tran G, Jabri B, Chatila TA, et al The toll-like receptor 2 pathway established colonization by a commensal of the human microbiota. Science 2011; 332:974–977. 10.1126/science.1206095 21512004 PMC3164325

[17] Mazmanian SK, Kasper DL. The love-hate relationship between bacterial polysaccharides and the host immune system. Nature Reviews 2006; 6:849–858. 10.1038/nri1956 17024229

[18] Mazmanian SK, Round JL, Kasper DL. A microbial symbiosis factor prevents intestinal inflammatory disease. Nature 2008; 453:620–625. 10.1038/nature07008 18509436

[19] Comstock LE, Coyne MJ, Tzianabos AO, Pantosti A, Oderdonk A, Kasper DL. Analysis of capsular polysaccharide biosynthesis locus of *Bacteroides fragilis*. Infect Immun 1999; 67:3525–3532. 10377135 10.1128/iai.67.7.3525-3532.1999 PMC116540

[20] Coyne MJ, Tzianabos AO, Mallory BC, Carey VJ, Kaper DL, Comstock LE. Polysaccharide biosynthesis locus required for virulence of *Bacteroides fragilis*. Infect Immun 2001; 69:4342–4350. 10.1128/IAI.69.7.4342-4350.2001 11401972 PMC98505

[21] Sanderson IR, Ezzell RM, Kedinger M, Erlanger M, Xu ZX, Pringault E, et al Human fetal enterocytes in vitro: modulation of the phenotype by extracellular matrix. Proc Natl Acad Sci USA1996; 93:7717–7722. 10.1073/pnas.93.15.7717 PMC38813 8755542

[22] Baumann H, Tzianabos AO, Brisson JR, Kasper DL, Jennings HJ. Structural elucidation of two capsular polysaccharides from one strain of *Bacteroides fragilis* using high-resolution NMR spectroscopy. Biochemistry 1999; 31:4081–4089. 10.1021/bi00131a026 1567854

[23] McCloy RA, Rogers S, Caldon CE, Lorea T, Castro A, Burgess A. Partial inhibition of Cdk1 in G 2 phase overrides the SAC and decoules mitotic events. Cell Cycle 2014; 13:1400–1412. 10.4161/cc.28401 24626186 PMC4050138

[24] Meng D, Newburg DS, Young C, Baker A, Tonkonogy SL, Sartor RB, et al Bacterial symbionts induce a FUT2-dependent fucosylated niche on colonic epithelium via ERK and JNK signaling. Am J Physiol Gastrointest Liver Physiol 2007; 293:G780–787. 10.1152/ajpgi.00010.2007 17673542

[25] Underwood MA, Arriola J, Gerber CW, Kaveti A, Kalanetra KM, Kananurak A, et al Bifidobacterium longum subsp. infantis in experimental necrotizing enterocolitis: alterations in inflammation, innate immune response, and the microbiota. Pediatr Res 2014; 76:326–333. 10.1038/pr.2014.102 25000347 PMC4167942

[26] Van Haver ER, Sangild PT, Oste M, Siggers JL, Weyns AL, Van Ginneken CJ. Diet-dependent mucosal colonization and interleukin-1beta responses in preterm pigs susceptible to necrotizing enterocolitis. J Pediatr Gastroenterol Nutr 2009; 49:90–98. 10.1097/MPG.0b013e31818de393 19516189

[27] Viscardi RM, Lyon NH, Sun CC, Hebel JR, Hasday JD. Inflammatory cytokine mRNAs in surgical specimens of necrotizing enterocolitis and normal newborn intestine. Pediatr Pathol Lab Med 1997; 17:547–559. 9211547

[28] Mogensen TH. Pathogen recognition and inflammatory signaling in innate immune defenses. Clin Microbiol Rev 2009; 22:240–273. 10.1128/CMR.00046-08 19366914 PMC2668232

[29] Gribar SC, Anand RJ, Sodhi CP, Hackam DJ. The role of epithelial Toll-like receptor signaling in the pathogenesis of intestinal inflammation. J Leukoc Biol 2008; 83:493–498. 10.1189/jlb.0607358 18160540

[30] Mazmanian SK, Liu CH, Tzianabos AO, Kasper DL. An immunomodulatory molecule of symbiotic bacteria directs maturation of the host immune system. Cell 2005; 122:107–118. 10.1016/j.cell.2005.05.007 16009137

[31] Noverr M, Huffnagle GB. The microflora hypothesis’ of allergic diseases. Clin Exp Allergy 2005; 35:1511–1520. 10.1111/j.1365-2222.2005.02379.x 16393316

[32] Claud EC, Lu L, Anton PM, Savidge T, Walker WA, Cherayil BJ. Developmentally-regulated IκB expression in intestinal epithelium and susceptibility to flagellin-induced inflammation. Proc Natl Acad Sci USA 2004; 101:7404–7408. 10.1073/pnas.0401710101 15123821 PMC409931

[33] Tzianabos AO, Pantosti A, Baumann H, Brisson J-R, Jennings AJ, Kasper DL. The capsular polysaccharide of *Bacteroides fragilis* comprises two ionically linked polysaccharides. J Biol Chem 1992; 267:18230–18235. 1517250

[34] Park BS, Song DH, Kim HM, Choio BS, Lee H, Lee JO. The structural basis of lipopolysaccharide recognition by the TLR4-MD-2 complex. Nature 2009; 20:458:1191–1195. 10.1038/nature07830 19252480

[35] Maeshima N, Fernandez RC. Recognition of lipid A variants by the TLR4-MD-2 receptor complex. Frontiers in Cell Infec Microbiol 2013; 3:1–13. 10.3389/fcimb.2013.00003 PMC3569842 23408095

[36] Cahill CM, Zhu W, Oziolor E, Yang YJ, Tam B, Rajanaia S, et al Differential expression of the activator protein 1 transcription factor regulates interleukin-1 β induction of interleukin 6 in the developing enterocyte. Plos One 2016; 11:e0145184 10.1371/journal.pone.0145184 26799482 PMC4723075

