# Supplementary material for: The symbiotic bacterial surface factor polysaccharide A on Bacteroides fragilis inhibits IL-1β-induced inflammation in human fetal enterocytes via toll receptors 2 and 4
Source: PLoS One. 2017 Mar 9;12(3):e0172738. doi: 10.1371/journal.pone.0172738 (PMC5344356; doi:10.1371/journal.pone.0172738)
Supplement: S1 File — (PDF) [file pone.0172738.s001.pdf]

Table A
The values used to build Fig 1

	IL-8 (pg/mg protein)
**Con**	285.86
	285.24
	286.29
**IL-1β**	11515.97
	11686.19
	14367.54
**PSA Dossa 1x10^7-IL-1β**	7462.89
	9976.29
	6915.41
**PSA Dossa 1x10^8-IL-1β**	8992.56
	7631.42
	6804.66
**PSA Delta 1x10^7-IL-1β**	13349.70
	11599.92
	11957.53
**PSA Delta 1x10^8-IL-1β**	13761.03
	11727.03
	10545.17
**PSA Dossa 1x10^8**	278.37
	353.48
	322.35
**PSA Dossa 1x10^7**	271.29
	283.57
	275.66
**PSA Delta 1x10^8**	284.19
	294.64
	284.20
**PSA Delta 1x10^7**	293.37
	298.71
	276.70

Table B
The values used to build Fig 2

IL-8 (pg/mg protein)			
**Con**	**IL-1β**	**PSA**	**PSA-IL-1β**
135.30	30390.01	552.78	11523.44
136.07	18989.04	603.13	12300.21
139.45	27151.80	641.32	9819.29

Table C<br>The values used to build Fig 3			
IL-8 (pg/mg protein)			
**Con**	**IL-1β**	**PSA**	**PSA-IL-1β**
263.68	27222.76	2698.95	12774.95
249.28	36011.37	3339.18	14359.13
307.60	52033.61	3999.62	17543.08

Table D<br>The values used to build Fig 4A			
IL-8 (pg/mg protein)			
**Con**	**IL-1β**	**PSA**	**PSA-IL-1β**
160.45	21391.19	309.40	13454.10
151.75	16404.09	283.51	11805.86
154.56	22703.12	276.68	11566.20

Table E<br>The values used to build Fig 4B			
IL-8 (pg/mg protein)			
**Con**	**IL-1β**	**PSA**	**PSA-IL-1β**
66.10	14314.22	184.81	19017.24
73.46	18462.72	232.60	27209.19
80.23	21964.54	193.78	15041.71

Table F<br>The values used to build Fig 4C			
IL-8 (pg/mg protein)			
**Con**	**IL-1β**	**PSA**	**PSA-IL-1β**
149.60	19444.92	296.32	19291.50
146.27	13193.91	267.07	13682.37
143.94	12868.16	304.54	19054.79

Table G<br>The values used to build Fig 5A			
IL-8 (pg/mg protein)			
**Con**	**IL-1β**	**PSA**	**PSA-IL-1β**
217.48	672.74	260.44	579.48
216.57	650.07	263.74	534.61
215.65	624.52	226.03	524.94

Table H<br>The values used to build Fig 5B			
IL-8 (pg/mg protein)			
**Con**	**IL-1β**	**PSA**	**PSA-IL-1β**
86.87	583.08	196.69	818.67
139.35	619.95	191.21	816.20
171.42	571.94	182.74	858.65

Table I<br>The values used to build Fig 5C			
IL-8 (pg/mg protein)			
**Con**	**IL-1β**	**PSA**	**PSA-IL-1β**
401.45	1009.95	653.71	1144.52
401.79	946.64	623.00	1028.24
402.47	961.41	619.37	1018.60

Table J<br>The values used to build Fig 6A2			
Corrected total cell fluorescence(CTCF)			
**Con**	**IL-1β**	**PSA**	**PSA-IL-1β**
240581.2	133112.6	52944.3	345339.9
253791.7	106915.3	21436.0	354798.8
122347.3	120098.1	17973.8	85206.1
166935.9	321021.2	38021.2	90981.9
215873.5	106294.0	25402.6	249631.4
202296.0	178044.8	26109.0	78372.3
215372.9	176969.5	46383.2	72197.2
199274.8	328552.5	19277.6	166829.6
187118.0	455295.8	53902.0	366060.1
146774.6	223065.5	74072.5	128872.0
234676.2	255676.4	47319.7	85327.5
290887.3	68302.1	152964.2	63426.5
207072.4	329828.3	38545.1	306926.2
104019.9	137638.0	119619.5	339009.9
180661.7	201532.3	31117.6	154193.3
132170.6	391165.2	21368.6	116064.8
296090.0	261261.3	41002.8	241077.7
171449.2	153456.0	57757.2	101104.6
122518.6	155881.5	44454.4	497558.7
175528.1	256073.0	88978.9	330760.3
78326.0	400975.6	143745.9	340608.3
82987.3	587258.8	154004.9	273334.3
134374.6	317071.7	50723.7	210760.5
235547.1	564579.8	61573.0	169631.6

94705.7	345964.8	33100.9	208604.9
91386.7	177422.5	55860.3	294315.1
140105.1	271173.9	22812.8	223131.0
115502.5	219938.8	79212.2	123469.4
153656.3	158307.3	83468.5	159047.7
155027.1	181295.4	385203.4	191459.6
153409.1	259917.7	102221.6	307603.7
257495.3	483816.4	77397.1	59126.5
133365.5	289390.8	53113.0	61198.8
161572.7	319690.0	34838.0	73422.9
290634.9	289443.5	49873.0	209828.7
167086.2	258187.8	58257.7	209350.3
361907.5	398752.5	36752.2	83609.6
215012.5	327361.4	33120.8	227355.7
269126.9	373642.2	65015.6	153445.1
122147.4	231275.5	36477.9	135045.4
36251.5	585216.3	89362.3	180888.8
157945.7	643179.2	39404.0	252464.7
86955.8	479616.8	23714.8	112274.9
95309.3	325843.4	133874.0	110819.9
184784.8	241282.7	60692.5	253980.7
90164.1	605553.1	43186.2	310586.6
53071.1	305996.6	71494.0	260333.2
54635.4	240364.2	82157.8	202770.3
35105.2	186671.4	92303.7	222349.6
	221256.8	184695.6	207231.7
	211819.1	62132.4	402248.6
	430274.4	61823.0	207783.7
	146237.4	24481.1	46006.1
	319942.8	51728.5	89691.9
	208164.0	37371.9	81962.5
	289138.5	43822.5	275313.0
	267683.2	40737.6	269353.2
	298502.3	172786.8	302887.4
	494033.2	118314.4	125890.9
	272216.2	63501.0	279920.0
	262103.3	63640.1	54899.8
	229011.4	65332.5	220060.1
	261756.9	118924.3	283418.4
	183450.9	84955.8	202830.7
	685807.2	70906.4	92570.9
	436150.9	384939.8	446616.8
	206923.9	68880.3	102741.2
	300778.8	37066.9	164692.6

	240871.4	32472.4	96524.6
	388352.4	60686.1	170784.2
	236207.5	85348.4	454644.1
	573460.8	69931.1	299064.2
	231129.9	79724.4	185823.2
	305002.3	62301.5	104411.6
	201667.5	92061.7	204841.7
	270841.4	114205.5	199633.2
	271503.1	147600.4	135776.5
	644691.6	14846.0	294406.4
	339069.2	30544.9	119037.0
	369090.9	94178.0	281841.8
	427915.2	118185.4	308179.1
	411747.1	228728.9	205414.1
	357215.1	56273.1	207299.3
	236096.6	118943.1	214023.6
	345124.6	48496.1	60859.7
	366849.7	27832.8	106054.2
	357014.3	112111.7	51424.9
	188193.2	105634.8	172992.9
	354748.0	139241.3	401513.6
	261692.8	96667.9	124841.4
	218986.4	25572.7	75555.0
	238290.8	21569.2	168549.8
	425302.1	21569.2	53235.5
			100219.1
			84463.3
			258714.5
			209515.0
			91638.3
			107526.0
			230812.3
			101710.3
			75206.2
			138062.2
			99731.3
			279332.9
			249163.7
			135759.8

Table K<br>The values used to build Fig 6B2			
Corrected total cell fluorescence(CTCF)			
**Con**	**IL-1β**	**PSA**	**PSA-IL-1β**
30588.4	172281.0	104920.5	193780.0
42080.2	156756.1	159683.0	236137.5
51639.2	175012.7	309771.1	318211.3
60909.8	284183.8	61318.4	170695.9
31291.7	153800.3	143373.4	164018.2
42434.2	224958.9	197757.5	235763.8
38068.8	223685.0	135407.7	124363.7
19449.1	240872.5	102849.6	163587.3
58062.0	209867.1	118367.8	151294.1
302719.8	180818.4	67170.8	239375.9
42804.8	188718.7	53138.4	193515.3
71009.2	248536.9	113316.4	240498.2
28795.8	230645.1	113658.8	123173.5
33754.7	298481.8	79547.0	91752.9
48791.8	125424.6	141287.1	212607.6
36275.4	299725.1	69692.5	269028.0
32314.2	515753.0	80600.8	260656.9
15787.5	92948.0	224695.8	317961.6
60575.8	119422.0	58173.9	224119.3
60575.8	201181.0	81721.0	207092.7
97299.5	178506.0	53995.3	270294.5
149579.4	209665.6	221229.6	90688.1
148978.4	167496.6	101356.3	170259.8
213905.4	144218.0	86645.9	102649.4
90705.0	381037.9	106758.4	81347.0
107136.8	204570.8	142557.2	140044.1
60642.5	272457.8	103927.7	104977.8
80312.6	131272.1	191728.7	171617.1
82900.5	286071.2	123051.3	238942.7
97271.4	82982.5	139443.1	208007.0
218462.8	148036.9	42919.9	191160.5
102093.6	136324.2	57098.3	256280.6
74177.1	257752.6	367081.1	191506.0
154600.0	291155.5	132230.4	172387.7
119999.4	173619.8	84334.7	137425.1
238375.1	104575.4	115581.9	140598.8
151069.1	181331.8	103364.1	239278.6
212077.1	118279.5	55512.6	159610.8
124052.0	34939.0	53849.4	87206.6

45366.9	139810.0	54323.3	139737.5
123053.7	73826.0	127858.2	225036.3
61737.4	149233.4	68329.3	293495.8
49205.1	188252.6	26764.4	117527.0
75290.8	183970.8	52079.3	117311.4
271545.5	290825.8	68293.4	367553.8
205735.5	147305.3	102539.8	175245.4
52086.5	206649.3	178706.0	308134.8
75119.2	115109.6	182080.9	163166.6
52038.0	173732.6	106620.1	258756.1
43942.0	205491.0	65639.3	261920.5
113708.7	341830.4	137751.2	325729.4
80905.1	322879.7	198914.5	463300.7
61910.9	264332.9	189552.9	629559.9
59524.6	314282.7	230422.8	287902.6
63169.1	557057.0	173533.2	236494.4
27432.2	272601.3	150435.8	219861.0
128449.0	116576.6	126612.8	392821.3
74760.0	268996.4	79070.7	235355.9
81933.2	245818.7	63488.9	451814.4
53692.8	170807.7	55036.8	175646.5
32076.7	178468.7		215664.3
132583.4	219776.1		204436.4
86773.1	232583.9		96806.9
57984.6	150675.4		89043.1
27538.3	268747.0		66132.9
63109.1	258602.6		36257.1
98333.8	249850.5		162056.2
80371.5	229171.0		182765.5
67586.2	189853.9		246385.9
67199.1	110031.4		315853.3
96248.9	160322.8		109954.0
38605.1	400113.1		125485.2
66194.1	156553.5		216410.9
57977.9	277041.6		265074.5
169351.9	160837.3		150164.6
256242.6	318827.4		124798.4
57128.3	164931.5		109155.7
105835.6	429404.1		367984.7
61772.9	199966.3		189815.4
50226.3	145529.4		218017.0
99074.6	341714.6		219546.9
106863.7	250370.6		80293.8
92317.7	145207.5		140735.3

73787.8	390664.2		258782.8
43249.4	385857.4		223084.0
144364.4	356768.7		295040.9
88462.8	252703.0		318917.5
	204505.9		223450.1
	351388.8		408999.3
	238180.8		274200.8
	219215.5		290813.2
	226400.8		99841.1
	183579.7		137811.3
	263191.7		127272.0
	290822.6		137871.1
	138917.5		103581.2
	278881.4		393341.8
	154981.6		532516.1
	226866.9		410948.4
	312708.1		322949.7
	116429.1		292331.0
	287170.7		463908.3
	274884.8		300836.5
	257694.8		247815.0
	205312.4		414881.5
	332655.0		
	210751.7		
	224619.8		
	192297.3		
	281692.8		
	186773.1		

**Table L<br>The values used to build Fig 6C2**			
**Corrected total cell fluorescence(CTCF)**			
**Con**	**IL-1β**	**PSA**	**PSA-IL-1β**
426069.2	126296.9	118420.7	201676.3
70041.5	179796.2	133993.8	93221.9
67485.2	303384.4	182906.2	171237.8
239152.1	300094.9	285265.7	109956.9
123230.5	409597.5	242911.3	131840.3
75642.5	350463.7	137774.2	247388.8
152338.3	156238.0	256070.0	238461.0
151957.9	294598.3	152030.5	184599.2
120653.3	259104.2	73635.0	182039.2
38284.6	124952.8	141103.4	337094.7
360282.1	264912.7	99675.0	143399.6

195903.5	477412.3	195598.0	851873.0
109842.9	134574.7	102338.8	440206.6
574010.0	249999.5	62843.5	241757.3
224792.5	219460.2	129552.8	127719.8
66161.0	306469.1	69548.5	114873.3
53642.5	175649.8	125452.3	264072.2
270938.3	753833.0	122530.0	350994.6
201995.5	260921.8	246747.7	237327.8
143508.2	643209.5	101040.3	80610.3
76729.5	371741.1	583558.0	226007.7
151211.6	771046.0	76145.5	483574.6
89763.4	369859.1	112646.9	194490.3
421239.4	253851.7	65617.5	377397.2
144897.2	199132.8	47269.6	368925.9
37811.9	240695.5	127578.8	108605.2
490920.8	263399.9	230732.3	181585.6
351157.3	686995.0	354648.4	159987.5
215975.8	447179.3	180559.7	265544.8
85847.9	300517.4	110240.3	190040.9
157339.7	392982.8	336433.2	213468.4
185736.7	165518.3	290336.7	99100.2
196824.0	411785.6	230967.3	127336.8
372660.0	213829.1	108408.8	172898.0
282898.4	301534.4	62467.9	264141.3
510300.5	305156.4	189521.1	299690.1
314941.7	295223.4	66957.7	175382.8
200076.0	580180.0	166928.0	136360.8
149382.3	164929.9	38959.1	124950.0
320357.7	224861.3	407037.0	266770.9
117302.3	496145.5	240068.0	329476.6
94064.4	339872.4	168343.0	400481.8
159697.7	440642.1	176694.7	421485.1
176942.2	626927.5	157237.7	117213.4
312549.1	281152.1	184415.0	135047.8
559932.5	425261.2		306358.3
89514.9	534237.0		207040.3
65232.8	338391.3		199273.3
214972.5	367302.7		339209.8
370647.7	561482.5		150476.0
143479.5			165839.0
